# Regurgitation under the ERAS Program: A Case Report

**DOI:** 10.3390/clinpract11040092

**Published:** 2021-10-17

**Authors:** Marta Pires, Margarida Marcelino

**Affiliations:** Instituto Português de Oncologia de Lisboa, 1099-023 Lisbon, Portugal; mmarcelino@ipolisboa.min-saude.pt

**Keywords:** regurgitation, ERAS, aspiration, carbohydrate drink

## Abstract

Introduction: Enhanced recovery after surgery (ERAS) is an evidence-based concept that reduces the recovery period after major abdominal surgery. Ingestion of carbohydrate solutions up until two hours before elective surgery has shown positive results. The authors present a case of regurgitation in a patient with apparently low risk for delayed gastric emptying who drank a carbohydrate solution two hours before induction of anaesthesia. Case report: An 80-year-old male patient with a relevant history of ischemic heart disease, atrial fibrillation, stage 3 chronic kidney disease and hypertension, was diagnosed with rectal cancer. He was scheduled for an anterior rectal resection hand-assisted laparoscopic surgery under the ERAS program, which included a 200 mL carbohydrate drink the night before and in the morning of the surgery, no less than two hours before the induction of anaesthesia. Immediately after loss of consciousness, there was regurgitation of a significant amount of clear fluid. Discussion: Even though ingestion of oral carbohydrate drinks is considered to be safe up to two hours before anaesthesia, further evaluation (e.g., gastric ultrasonography) may be considered in non-high-risk patients.

## 1. Introduction

The concept of enhanced recovery after surgery (ERAS) was introduced in 2001 and has since then been gradually gaining evidence support through numerous scientific studies. Its main goal is to achieve minimum loss of patient functional capacity and improve the recovery period after major abdominal surgery [1].

Amongst many others, one of its recommendations concerns preoperative fasting. In compliance with fasting guidelines of various anaesthesiology societies, it suggests that patients scheduled for elective surgery should be encouraged to ingest complex carbohydrate solutions up until two hours before the induction of anaesthesia [1,2,3].

This should increase intraoperative insulin sensitivity, reduce postoperative insulin resistance, decrease protein breakdown and reduce the recovery period. The possibility of delayed gastric emptying must be taken into account before deciding the best approach for a specific patient [1].

We present a case of regurgitation during induction of anaesthesia in a patient under the ERAS program.

## 2. Case Report

An 80-year-old Caucasian male patient diagnosed with stage cT2/T3N1 rectal adenocarcinoma with neoadjuvant radiotherapy was scheduled for an anterior rectal resection hand-assisted laparoscopic surgery under the ERAS program. 

The patient’s medical history included severe ischemic heart disease that required 4 drug-eluting stents after a STEMI event 12 months before this surgery and heart failure (ejection fraction of 52%) in NYHA Class I, permanent atrial fibrillation, stage 3 chronic kidney disease diagnosed 3 years earlier, hypertension, benign prostatic hyperplasia, and glaucoma. Patient’s medication included aspirin 100 mg id, furosemide 40 mg id, ramipril 1.25 mg id, bisoprolol 1.25 mg id, rosuvastatin 20 mg id, apixaban 2.5 mg id, tansulosine 0.4 mg id, finasteride 5 mg id, esomeprazole 40 mg id, and eye drops of brimonidine and tafluprost. His body mass index was 24.86 kg m^−2^ and relevant laboratory evaluation included HbA1c 5.5%, creatinine 1.12 mg/dL and urea 49 mg/dL. There was no history of gastroesophageal reflux or hiatal hernia. 

He was admitted the day before surgery and started the institution’s ERAS protocol, which includes a 200 mL iso-osmolar, non-carbonated, 0.5 kcal/mL carbohydrate drink the night before and in the morning of the surgery, no less than 2 h before induction of anaesthesia. Bowel preparation included polyethylene glycol-electrolyte solution and metronidazole as per the institution’s protocol.

On the day of surgery, he was asymptomatic and received no pre-anaesthetic medication. After placing American Society of Anaesthesiologists (ASA) standard monitors and pre-oxygenation, general anaesthesia was induced with fentanyl 2 mcg kg^−1^, propofol 2 mg kg^−1^ and rocuronium 0.6 mg kg^−1^. Immediately after loss of consciousness, there was regurgitation of a significant amount of clear fluid. The patient was placed in Trendelenburg position, the oropharynx was suctioned, and an endotracheal tube was placed and suctioned before starting positive-pressure ventilation.

Despite this incident, there were no signs of respiratory distress and, after discussion with the surgeon, the decision was to proceed with surgery. Throughout the procedure, there were no signs of bronchospasm, increased airway pressure or desaturation. At the end of the surgery, flexible bronchoscopy revealed no damage or content in the airway. The surgery was otherwise successful, a postoperative chest radiograph showed no alterations compared with the preoperative image and no prophylactic treatment for aspiration pneumonia was introduced. The patient recovered uneventfully and was discharged at the third postoperative day.

## 3. Discussion

In order to reduce the risk of pulmonary aspiration of gastric content during anaesthesia induction, a period of preoperative fasting is generally recommended. According to the ASA fasting guidelines, carbohydrate drinks are considered clear fluids that may be ingested up to two hours before anaesthesia. However, there is equivocal evidence for gastric volume at fasting times of two to four hours and there is no clear statement of how much volume is safe [2].

In addition, consensus statements for ERAS in gastrointestinal surgery do not recommend preoperative treatment with oral complex carbohydrates in patients with documented delayed gastric emptying or gastrointestinal motility disorders [1] and, to our knowledge, no regurgitation event in ERAS patients has been reported. 

Known patient risk factors for delayed gastric emptying are obesity, diabetes *mellitus*, history of gastroesophageal reflux disease and opiate analgesic therapy [4]. Chronic kidney disease is a known but frequently unmentioned risk factor for delayed gastric emptying [5] and evidence of which stage impacts gastric emptying is lacking.

Sharma et al. conducted a clinical audit of ASA fasting guidelines using gastric ultrasonography and found that risk factor association has a greater impact on residual gastric volume than hours of fasting. Interestingly, 30% of patients with chronic renal disease had high residual gastric volume [6]. Okabe et al. suggests that liquid gastric emptying is more dependent on total caloric content than on volume or even compositional differences [7].

Despite not being available in most centres, gastric emptying tests are gold standard to diagnose gastroparesis. Ultrasound assessment of preoperative gastric volume appears to be an effective screening tool in patients with risk factors [4]. Although it requires specific technical skills and equipment, which limits its widespread use, there is a favourable learning curve with appropriate training [8].

In patients with high risk of pulmonary aspiration of gastric content, preventive interventions, such as the use of antihistamines and/or antacids, 30–45° tilt of the upper body, nasogastric tube placement, rapid sequence induction or cricoid pressure, are widely accepted. However, these manoeuvres have their own risks, and their effectiveness is debatable [9]. In non-high-risk patients, such as the case presented, further risk assessment with gastric ultrasonography may assist the decision-making process [10,11]. 

Other than an asymptomatic chronic kidney disease, this patient presented with no risk factors for regurgitation of gastric content that would help predict this incident. Even though ingestion of oral carbohydrate drinks is considered to be safe up to two hours before induction of anaesthesia, it should be taken into consideration during the individual assessment of each patient and further evaluation (e.g., gastric ultrasonography) may be considered.
